# Acute Flaccid Myelitis Associated with Enterovirus D68 in Children, Argentina, 2016

**DOI:** 10.3201/eid2503.170897

**Published:** 2019-03

**Authors:** Carolina M. Carballo, Marcela García Erro, Nora Sordelli, Gabriel Vazquez, Alicia S. Mistchenko, Claudia Cejas, Manlio Rodriguez, Daniel M. Cisterna, Maria Cecilia Freire, Maria M. Contrini, Eduardo L. Lopez

**Affiliations:** Hospital de Niños “Ricardo Gutiérrez,” Buenos Aires, Argentina (C.M. Carballo, M. García Erro, N. Sordelli, A.S. Mistchenko, M. Rodriguez, M.M. Contrini, E.L. Lopez);; Fundación para la Lucha contra las Enfermedades Neurológicas de la Infancia (FLENI), Buenos Aires (G. Vazquez, C. Cejas);; Administración Nacional de Laboratorios e Institutos de Salud (ANLIS) “Dr. Carlos G. Malbrán,” Buenos Aires (D.M. Cisterna, M.C. Freire)

**Keywords:** enterovirus, infectious disease outbreaks, myelitis, children, acute flaccid myelitis, Argentina, viruses, EV-D68

## Abstract

After a 2014 outbreak of severe respiratory illness caused by enterovirus D68 in the United States, sporadic cases of acute flaccid myelitis have been reported worldwide. We describe a cluster of acute flaccid myelitis cases in Argentina in 2016, adding data to the evidence of association between enterovirus D68 and this polio-like illness.

We report a cluster of acute flaccid myelitis (AFM) cases in Buenos Aires, Argentina, in 2016. AFM was defined as acute flaccid paralysis (AFP) with magnetic resonance imaging (MRI) showing lesions predominantly affecting the gray matter of the spinal cord (*1*). We prospectively studied all patients with AFP who were admitted to Hospital de Niños “Ricardo Gutiérrez” in Buenos Aires during April 24–August 24, 2016, under the Argentine National Surveillance Acute Flaccid Paralysis Program for poliovirus as part of the World Health Organization AFP Program in the Americas. We obtained fecal samples or rectal swab specimens, serum samples, nasopharyngeal swab specimens, and cerebrospinal fluid (CSF) samples.

Fecal samples were tested at the National Reference Center for the Argentine National Surveillance Acute Flaccid Paralysis Program for enterovirus, including wild-type and vaccine-derived poliovirus. We screened clinical samples for enterovirus D68 (EV-D68) using a panrhinovirus and enterovirus nested PCR of enterovirus targeting the 5′ untranslated region (*2*). We purified the amplified products and prepared them for Sanger sequencing. We performed BLAST searches (https://blast.ncbi.nlm.nih.gov/Blast.cgi) of GenBank sequences to identify which picornavirus was present. We obtained viral protein 1 partial sequences as previously described (*3*). In addition, we studied a wide panel of viruses (parainfluenza virus 1, 2, and 3; influenza A/B; respiratory syncytial virus; adenovirus; metapneumovirus; rhinovirus; varicella zoster virus; herpes simplex virus; cytomegalovirus) by reverse transcription PCR (RT-PCR) and studied bacteria by culture. We performed MRI and electromyography for all patients.

Fourteen children were admitted with AFP during April–August 2016. Six were confirmed to have AFM by case definition; the other 8 had alternative diagnoses, including Guillain-Barré syndrome (*3*), influenza virus myositis (*2*), encephalitis by echovirus (in 1 child with Down syndrome), acute transient hip synovitis (*1*), and transverse myelitis (*1*). Patients’ clinical, demographic, and outcome findings are shown in Table 1, diagnostic findings in Table 2.

**Table 1:** Demographics, neurologic symptoms, and clinical outcomes for patients with acute flaccid myelitis, Argentina, 2016

Feature	Patient 1	Patient 2	Patient 3	Patient 4	Patient 5	Patient 6
Age, mo/sex	34/M	15/F	35/M	60/F	12/F	60/F
History of asthma	No	No	No	Yes	Yes	Yes
Preceding illness						
Fever	No	Yes	Yes	Yes	Yes	No
URTI	Yes	Yes	Yes	Yes	Yes	Yes
Gastrointestinal symptoms	No	No	Yes	No	No	No
Neurologic symptoms						
Limb, back, or neck pain	Yes	Yes	Yes	Yes	Yes	Yes
Arm weakness	Yes (bilateral)	Yes (right)	No	Yes (left)	Yes (bilateral)	Yes (bilateral)
Leg weakness	Yes (bilateral)	Yes (progressive, asymmetric, bilateral)	Yes (left progressive to bilateral, asymmetric)	Yes (progressive, asymmetric, bilateral)	Yes (bilateral)	Yes (bilateral)
Neck weakness	Yes	Yes	No	Yes	Yes	Yes
Facial weakness	No	No	No	Yes	No	Yes
Sensitivity involvement	No	No	No	No	No	No
Mental status involvement	No	No	No	No	No	No
Other neurologic deficits	Bulbar weakness	No	No	Left VII cranial nerve palsy	No	Bilateral VII cranial nerve palsy; bulbar weakness; tetraparesis
Severity of disease	ICU care; mechanical ventilation; tracheostomy; feeding support	Weakness	Weakness	ICU care; noninvasive positive pressure ventilation; feeding support	Progressive asymmetric 4- limb weakness	ICU care; mechanical ventilation; tracheostomy; feeding support
Outcome/sequelae	Persistent weakness; feet atrophy; equinus left foot; chronic noninvasive ventilation support	Partial recovery of weakness Atrophy of left foot	Recovery of right leg weakness; equinus left foot	Persistent leg left paralysis; 2 cm atrophy in left quadriceps	Persistent left arm paralysis and left leg weakness	Persistent leg paralysis and arm weakness; noninvasive ventilation support
Duration of hospitalization	6 mo	14 d	10 d	46 d	8 d	4 mo

*ICU, intensive care unit; URTI, upper respiratory tract infection.

**Table 2:** Diagnostic findings in patients with acute flaccid myelitis, Argentina, 2016

Laboratory tests	Patient 1	Patient 2	Patient 3	Patient 4	Patient 5	Patient 6
Cerebrospinal fluid findings						
Leukocytes/mm^3^ (% mononuclear cells)	195 (85)	4 (100)	23 (84)	130 (96)	40 (70)	16 (54)
Glucose, mg/dL, reference range 40–70	53	58	60	55	57	76
Protein, mg/dL, reference range 15–50	41	70	33	34	41	34
Albuminocytological dissociation	No	Yes	No	No	No	No
Virologic findings						
Enterovirus-D68	Yes	Yes	Yes	Yes	No	No
Nontypable enterovirus	No	No	No	No	No	Yes
Type of positive specimen						
Nasopharyngeal aspirate	Yes	Yes	Yes	Yes	No	Yes
Feces	No	Yes	No	Yes	No	No
Cerebrospinal fluid	No	No	No	No	No	No
Time from prodromal illness to specimen collection	5 d	30 d	13 d	6 d	25 d	3 d

In 4 (66.7%) of 6 patients, we confirmed EV-D68 infection by nested RT-PCR. In 1 patient, enterovirus was detected but not typed; in 1 patient, no agent was detected. All patients had distinctive neuroimaging changes. We followed confirmed AFM cases for 6 months to assess clinical improvement.

The median age of patients with AFM was 3.9 (range 1–5) years; 4 (66.7%) of the 6 were female, and 3 (50%) had a history of asthma. All patients had prodromal signs or symptoms before onset of neurologic symptoms: 100% had upper respiratory tract infection (URTI); 4 (66.7%) had fever; and 1 (16.7%) had vomiting and abdominal pain. Neurologic symptoms appeared 1–11 (median 2) days after URTI symptoms. 

Results of hematology and chemistry analysis were normal for 5 (83%) patients. Patient 1 had leukocytosis (leukocytes 18,000 cells/mm^3^, with 82% neutrophils) and elevated levels of alanine aminotransferase (103 IU/L [reference 10–43 IU/L]), aspartate aminotransferase (97 IU/L [reference 10–35 IU/L]), and creatine kinase (6,591 IU/L [reference 24–170 IU/L]). During follow-up, patient 1 showed an increased creatine kinase level that could not be related to enterovirus infection. 

All confirmed AFM case-patients showed T2 gray matter hyperintensity within the spinal cord on MRI. Electromyography showed early signs of denervation and low motor neuron function in all 5 patients in whom the test could be done. Specimen collection was performed 9.5 (range 3–30) days after URTI symptoms started and 7.5 (range 1–18) days after onset of neurologic symptoms.

We identified enterovirus using nested RT-PCR of nasopharyngeal samples in 5 (83%) of 6 patients; 4 (80%) of 5 were typed as EV-D68, but in 1 patient (20%) the viral load was too low for typing. We identified EV-D68 in 2 (33%) of 6 fecal specimens. We performed molecular characterization of EV-D68 strains based on phylogenetic analyses of a partial VP1 genomic region (Figure).

**Figure:**
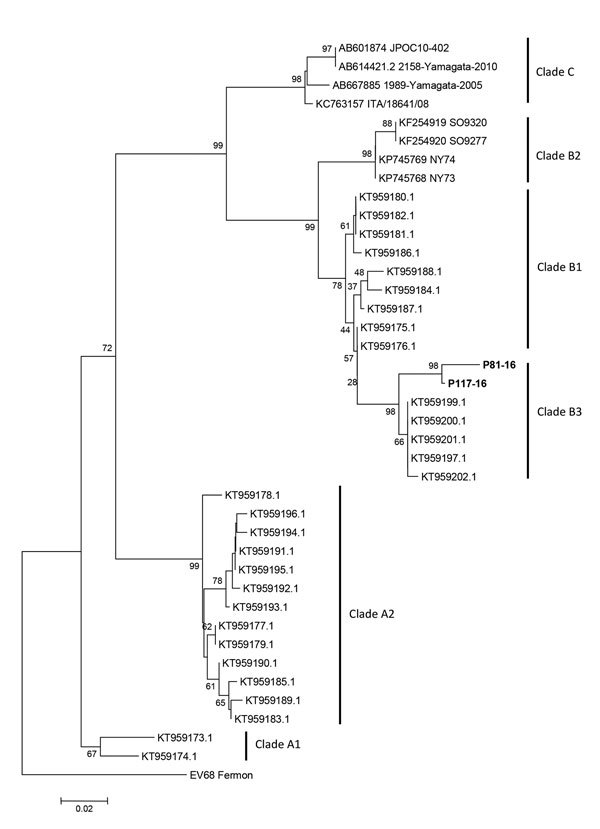
Molecular characterization of enterovirus D68 strains from Argentina, 2016, compared with reference strains from GenBank. Tree based on phylogenetic analyses of partial viral protein 1 genomic region (nucleotide positions 2554–2799, corresponding to the Fermon strain). Bold indicates strains detected in this study (GenBank accession nos. MF445419–20). We generated trees using the neighbor-joining method, as implemented in MEGA 6 software (http://www.megasoftware.net). Bootstrap values from 1,000 replicates are shown at the nodes. The trees were rooted with the prototype strain Fermon (GenBank accession no. AY426531). Scale bar indicates nucleotide substitutions per site.

Results of nested RT-PCR for enterovirus were negative for all CSF samples; results of the respiratory virus panel were negative for all patients. Neither bacteria nor fungus were isolated in blood or CSF samples. Serum PCR to identify herpes simplex virus, varicella zoster virus, and cytomegalovirus also yielded negative results.

Intravenous immunoglobulin was empirically infused in 5 (83%) patients; 2 (33%) received systemic corticosteroids. Three patients required intensive care unit admission. All patients had neurologic sequelae: persisting palsy in >1 limbs and atrophy of muscles with a shortening of limbs. Two patients required chronic noninvasive ventilatory support during 6 months of follow-up. No patients died.

## Conclusions

AFM has been associated with different etiologic agents (*1*). EV-D68 is a nonpolio enterovirus characterized by affinity for α2–6-linked sialic acids typically found in the upper respiratory tract, making the respiratory tract the preferred target for EV-D68 replication, unlike most enteroviruses, which replicate in the gut (*1**,**4*). Although there is no definitive evidence of causality between EV-D68 and AFM, since the 2014 EV-D68 respiratory outbreak in North America, AFM cases possibly associated with EV-D68 have been reported in the United States, Canada, Australia, Norway, Great Britain, and France (*1**,**5*). We report a cluster of AFM associated with EV-D68 in Argentina; another institution in Argentina (Hospital Garrahan) has also reported a case series of AFM (*6**,**7*).

The cluster in this report occurred over a 3-month period, during the 2016 autumn–winter season, which is the typical enterovirus season in Buenos Aires. Clinical and neurologic findings were similar to those of cases reported in other countries, including URTI preceding the neurologic features (*4**,**8**,**9*). Patients were admitted with asymmetric, acute, and progressive weakness of limbs; areflexia; and muscle pain. These symptoms have been reported as polio-like syndrome; however, testing and MRI should be performed for multiple viruses, including enteroviruses and EV-D68, to detect distinctive spinal cord lesions. No sensory sensitivity involvement was observed. Two patients had cranial nerve dysfunction. Laboratory findings were similar to those previously described, including CSF abnormalities (*1**,**4**,**8*).

Different hypotheses to explain difficulties in isolation of EV-D68 have been reported (*4*). It is possible that most of the nasopharyngeal specimens in previous studies and in our cluster were taken after 7 days of URTI, when the viral load is usually low, as reported by Imamura et al. (*10*). In our case series, enterovirus was identified in respiratory secretions in 5 (83.3%) of 6 patients, even though specimen collection was performed >7 days (mean 9 days) after AFM onset (in 1 patient, viral load was too low for genotyping). The negative nasopharyngeal specimen was collected at 18 days after onset.

Isolation of EV-D68 in fecal samples is uncommon because the virus is both heat and acid labile (*1*). However, in 2 (33.3%) of our 6 patients, EV-D68 was identified in fecal samples.

Reported rates of CSF detection of known neurotropic enteroviruses, such as polioviruses and enterovirus A71, are as low as 0%–5%, although viruses could be detected in brain or spinal cord tissue (*4**,**11*). A recent mouse model of AFM caused by EV-D68 showed that EV-D68 infects anterior horn motor neurons, resulting in motor neuron death (*9*). In our series, CSF samples tested negative for EV-D68 and other pathogens.

No specific treatment for EV-D68 AFM is available; the US Centers for Disease Control and Prevention recommends only support measures (*7**,**12*). Zhang et al. demonstrated that commercial immunoglobulin contained high levels of neutralizing antibodies against EV-D68 strains during the 2014 outbreak in the United States (*13*). No vaccines are available.

EV-D68 belonging to subclade B3 was identified in our cluster by molecular sequencing. This subclade was associated with EV-D68 circulation in the United States and Europe in 2016 (*14*). 

We show a cluster of AFM associated with EV-D68 in Argentina. Our findings contribute to global evidence of EV-D68 as a possible cause of localized polio-like illness.
